# Supplementary material for: Crystallization of Long-Spaced Precision Polyacetals III: Polymorphism and Crystallization Kinetics of Even Polyacetals Spaced by 6 to 26 Methylenes
Source: Polymers (Basel). 2021 May 13;13(10):1560. doi: 10.3390/polym13101560 (PMC8152236; doi:10.3390/polym13101560)
Supplement: Supplementary file 1 [file polymers-13-01560-s001.zip › polymers-1232057-supplementary.pdf]

# Crystallization of Long-Spaced Precision Polyacetals III: Polymorphism and Crystallization Kinetics of Even Polyacetals Spaced by 6 to 26 Methylenes

Stephanie F. Marxsen[1], Manuel Häußler[2], Stefan Mecking[2], and Rufina G. Alamo[1,*]

[1] FAMU-FSU College of Engineering, Department of Chemical and Biomedical Engineering, 2525 Pottsdamer St, Tallahassee, FL, 32310
[2] Department of Chemistry, University of Konstanz, Universitätsstraße 10, 78457 Konstanz, Germany

## Supporting Information

**Table SI.1: Level of crystallinity, long period, crystal thickness, and equilibrium melting temperature data for even polyacetals.**

| Sample | Crystal Type | $T$ (°C) [a] | $X_c$ [b] | $L = 2\pi/q$ (Å) [c] | $l_c$ (Å) [d] | $T_m^{\circ}$ (°C) [e] |
|---|---|---|---|---|---|---|
| PA-6 | Hexagonal + Form I | -10 | 0.363 | 102 | 38 | 32 |
| | Hexagonal + Form I | 0 | 0.389 | 105 | 38 | 32 |
| | Hexagonal + Form I | 10 | 0.381 | 112 | 41 | 32 |
| | Form I | 20 | 0.349 | 146 | 49 | 32 |
| PA-12 | Hexagonal + Form I | 0 | 0.453 | 84.1 | - | 65.5 |
| | Hexagonal + Form I | 25 | 0.438 | 88.3 | 33.5 | 65.5 |
| | Form I | 50 | 0.536 | 94 | 51 | 65.5 |
| | Form II | 60 | 0.533 | 110 | 59 | 71.5 |
| PA-18 | Disordered | 0 | - | 83.3 | 42.3 | 78 |
| | Hexagonal | 50 | 0.571 | 94.0 | 57.3 | 78 |
| | Form I | 67 | 0.662 | 113 | 67 | 78 |
| | Form II | 77 | 0.657 | 138 | 93 | 85.3 |
| PA-26 | Disordered | 0 | - | 73.9 | - | 85.5 |
| | Hexagonal | 70 | 0.563 | 105 | 55 | 85.5 |
| | Form I | 75 | 0.657 | 136 | 94 | 85.5 |
| | Form II | 80 | 0.655 | 167 | 101 | 95 |

[a] Crystallization or quenching temperature, [b] Level of crystallinity estimated from WAXD, [c] long period from Lorentz-corrected SAXS data, [d] lamellar thickness from the normalized one-dimensional correlation function, [e] Equilibrium melting temperature.
